# Dual sensory impairment: The association between glaucomatous vision loss and hearing impairment and function

**DOI:** 10.1371/journal.pone.0199889

**Published:** 2018-07-06

**Authors:** Lucy I. Mudie, Varshini Varadaraj, Prateek Gajwani, Beatriz Munoz, Pradeep Ramulu, Frank R. Lin, Bonnielin K. Swenor, David S. Friedman, Nazlee Zebardast

**Affiliations:** Johns Hopkins University, Baltimore, MD, United States of America; Moorfields Eye Hospital NHS Foundation Trust, UNITED KINGDOM

## Abstract

**Background:**

Hearing impairment, vision impairment, and dual impairment (both hearing and vision impairment), have been independently associated with functional and cognitive decline. In prior studies of dual impairment, vision impairment is generally not defined or defined by visual acuity alone. Glaucoma is a leading cause of blindness and does not affect visual acuity until late in the disease; instead, visual field loss is used to measure vision impairment from glaucoma.

**Objective:**

To examine the effect of glaucomatous visual field loss and hearing impairment on function.

**Design:**

Cross-sectional.

**Setting:**

Hospital-based clinic in Baltimore, Maryland.

**Subjects:**

220 adults, ≥55 years presenting to the glaucoma clinic.

**Methods:**

Vision impairment was defined as mean deviation on visual field testing worse than -5 decibels in the better eye, and hearing impairment was defined as pure tone average worse than 25 decibels on threshold audiometry testing in the better ear. Standardized questionnaires were used to assess functional status.

**Results:**

Five participants were excluded for incomplete testing, leaving 32 with vision impairment only, 63 with hearing impairment only, 42 with dual impairment, and 78 controls with no hearing impairment or vision impairment. Participants with dual impairment were more likely to be older and non-White. Dual impairment was associated with significantly more severe driving limitation and more difficulty with communication compared to those without sensory impairment when adjusted for age, race, gender and number of comorbidities.

**Conclusion:**

Older individuals with glaucoma and hearing loss seem to have generally poorer functioning than those with single sensory loss. Health professionals should consider visual field loss as a type of vision impairment when managing patients with dual impairment.

## Introduction

Sensory impairment is common; in 2010, 32.4 million people worldwide were blind, and 191 million had moderate to severe visual impairment (VI).[1] Moreover, the global prevalence of disabling hearing impairment (HI) was reported as 360 million in 2011.[2] In the United States of America, data from the 1999–2002 National Health and Nutrition Examination Survey (NHANES) showed that 14 million people 12 years or older were visually impaired in their better-seeing eye[3], while data from the 2001–2008 NHANES reported that 30 million people 12 years or older had bilateral HI and 48.1 million had unilateral HI.[4] Dual Impairment (DI) is the presence of both VI and HI. In a sample from the 1999–2006 NHANES, the prevalence of DI was 11% among adults over 80 years.[5] As the US population ages, the number of individuals with DI is expected to increase further as the proportion of adults 65 years of age and older reaches 18% by 2020.[6]

Both VI and HI affect every day functioning. VI has been linked to physical disability, physical decline[7,8], cognitive impairment[9], and increased mortality.[8,10] While HI has been associated with declines in physical[11] and cognitive functioning[12], DI has also been linked to decreased physical activity[13], lower cognition[9,14], impaired communication, social isolation, increased hospitalization, and greater difficulty driving including increased likelihood of motor vehicle crashes.[5, 15–17]

Prior DI research has examined the effect on functioning, but these studies have used a narrow definition of VI. In most studies of DI, VI is defined by visual acuity (VA), a measure of central vision ability, however this does not take into account VI from visual field (VF) loss. VI from glaucoma is distinct from other causes of VI and leads to VF loss while preserving VA. Glaucoma is responsible for 2% of VI and 8% of blindness worldwide.[18] By age 70, 2.1% of Americans have glaucoma, and this increases to 8% by 80 years of age.[19] Glaucoma tends to cause loss of peripheral vision first, rather than affect central VA, and often the patient is unaware of their vision loss until late in the disease. Disability associated with glaucoma-related VI is significant and affects several areas of functioning, with previous studies showing that glaucoma patients are more likely to restrict driving[20], report more limitations in activities of daily living[21] and fear of falling.[22]

While HI, VI from glaucoma, and DI are independently associated with functional and cognitive decline, there is conflicting evidence on whether DI results in greater difficulty with physical function, cognitive function, and worse mental health than VI or HI alone.[23, 24] Additionally, to our knowledge, no prior studies have examined the role of glaucomatous VF loss as the cause of VI in DI. In this study of glaucoma and HI, we hypothesized that older individuals with VI from glaucoma and concurrent HI would be more likely to report difficulties with physical functioning and communication, perform poorer on standardized tests of cognition, and have worse mental health than those who have single sensory impairment. Understanding the combined effect of VI from glaucoma and HI on cognitive and physical functioning may help to optimize evaluation and management of individuals with DI.

## Methods

The study was approved by the Johns Hopkins School of Medicine Institutional Review Board, and was conducted in compliance with the Health Insurance Portability and Accountability Act (HIPAA) and the Declaration of Helsinki. Informed consent was obtained from all participants prior to study entry.

### Study population

In 2012, two hundred and twenty participants were enrolled from the glaucoma clinic at the Wilmer Eye Institute, Baltimore. Glaucoma cases and controls aged 55–85 years who were able to communicate in English were recruited during their routine clinical visit. Subjects were included as glaucoma patients if they had primary open angle glaucoma, primary angle closure glaucoma, pseudoexfoliation glaucoma, or glaucoma from pigment dispersion syndrome. Participants had to have completed at least 2 VF tests with the more recent test having been performed within the previous 12 months. If the latter criterion was not met, VF testing was performed on the day of enrollment. Medical history questionnaires, hearing and grip strength testing were completed on the day of enrollment. 201/220 (91%) participants completed the functional status questionnaires in person on the day of enrollment, and the remaining subsequently completed them via phone.

### Evaluation of vision and hearing

VF examination was conducted using the Zeiss Humphrey Field Analyzer II 750i (Carl Zeiss Meditec Inc., Dublin, CA) following the Swedish Interactive Thresholding Algorithm (SITA) Standard 24–2 protocol.VI was defined as VF mean deviation (MD) worse than -5 decibels (dB) in the better eye on the most recent VF test. If the most recent VF test was unreliable (false positives or false negatives >20%), the previous VF test was used. If the previous VF test was also unreliable, the participant was excluded. Visual acuity using the Early Treatment of Diabetic Retinopathy chart (Innova Systems, Burr Ridge, IL)) and contrast sensitivity using the Pelli-Robson Acuity chart (Clement Clarke International, Essex, UK) was also measured for all participants.

Bilateral otoscopy was performed by trained research staff prior to hearing testing. Pure tone air-conduction audiometry was performed using ER3 insert earphones in a sound-attenuating booth (Interacoustics AD629 Diagnostic Audiometer; ETS-Lindgren, Cedar Park, TX) that met prevailing American National Standards Institute criteria (ANSI S3.6–1996). HI was defined as four-frequency (0.5–4 kHz) pure tone average (PTA) threshold worse than 25 dB in the better ear. DI was defined as presence of both VI and HI.

### Other tests and questionnaires

Participants underwent grip strength testing with a hydraulic dynamometer (PC5030J1, Fit Systems Inc., Calgary, Canada), and self-reported functional status evaluation using standardized questionnaires conducted by trained interviewers. The Geriatric Depression Scale (GDS)[25] Short Form was used to assess depression; this shorter version of the GDS consists of 15 items, and overall scores range from 0 to 15, with a score >5 suggesting depression, and >10 suggesting severe depression. The Mini-Mental State Examination (MMSE) for the visually impaired (MMblind)[26] was used to assess cognition; this test is identical to the original MMSE[27] test, but omits 8 items involving image processing, with a maximum possible score of 22, and a score <16 suggesting cognitive impairment.

Noise exposure history, use of hearing aids, Revised Quantified Denver Scale of Communication Function (QDS)[28], and Hearing Handicap Inventory in the Elderly Screening form (HHIE-S)[29] were used to assess self-reported hearing and communication impairment. The revised QDS consists of 5 items measuring self-reported communication function and is scored from 5 to 25, with scores >11 indicating some difficulty in communication. The HHIE-S consists of 10 items, and is scored from 0 to 40, with scores >10 indicating moderate probability of emotional and social difficulty due to hearing loss, and scores >25 indicating a high probability of emotional and social difficulty due to hearing loss.

Social functioning was assessed using the Social Network Index (SNI)[30], UCLA Loneliness scale[31], and Supplement on Aging (SOA-II) Social Component Questionnaire.[32] The SNI measures social network diversity and is scored from 0 to 12; with scores <3, 4–5, and 6+ indicating low, moderate, and high social network diversities, respectively. The UCLA Loneliness scale consists of 20 items scored from 20–80; a score of 20–34 indicates low levels of loneliness, and scores of 35–80 indicate moderate to high loneliness. The SOA-II consists of 9 items and is a qualitative evaluation of subjective attitudes towards level of social activity within the last 2 weeks.

The Driving Limitation Questionnaire[20] was also administered. This measures driving habits and vision-attributable driving limitations. It consists of 23 items and is scored from 0 to 9, with scores of 0–2, 2–3, and 4–9 indicating no, moderate, and severe driving limitations, respectively. Lastly, the Activities of Daily Living Questionnaire (ADL), and Intermediate Activities of Daily Living Questionnaire (IADL) from the Functional Status Questionnaire (FSQ)[33] were administered for further assessment of functional status. The self-reported number of ADLs or IADLs with any difficulty, or the number with severe difficulty were calculated for this analysis.

A medical history questionnaire was also administered to assess the presence of over 23 comorbidities from history of hip fracture to a diagnosis of vertigo. The total of number of comorbidities were added up for analyses.

### Statistical analysis

Participants were categorized as having either 1) no sensory loss, 2) VI only, 3) HI only, or 4) DI. Descriptive statistics for demographics and responses to questionnaires were calculated for each group. Analysis of variance and chi-squared tests were used to report unadjusted differences between groups, and multinomial logistic regression was used to report age-adjusted differences between groups. Box plots were made to illustrate the range of VI (as measured by VF better eye MD), and HI (as measured by better ear PTA threshold) among the groups.

Multivariable linear, logistic and negative binomial regressions were performed as appropriate to measure the association between VI, HI and DI as compared to no sensory impairment with self-reported functional status (as measured by different questionnaires), mood and cognition. Age, gender, race and number of comorbidities (0, 1, 2, 3+) were adjusted for in each model. All analyses were conducted in Stata v.14 (StataCorp LP, College Station, TX).

## Results

Five of the 220 recruited participants were excluded due to incomplete or unreliable VF, or hearing testing. 32 participants had VI only, 63 had HI only and 42 had DI. Participants with HI were significantly older than those without HI; the mean ages of participants with no sensory loss, VI only, HI only, and DI were 68.2 (Standard Deviation, SD: 4.9) years, 69.1 (SD: 5.3) years, 72.2 (SD: 5.6) years, and 73.6 (4.6) years, respectively (**Table 1**, p < 0.05). A greater proportion of HI participants identified as White compared to VI participants (81% vs. 56% respectively). Although 55% of participants with DI identified as White, the other groups had even higher proportions of Whites they were more likely to be non-White (p<0.01) than those without DI. There were no significant differences between groups in other characteristics **(Table 1)**.

**Table 1:** Characteristics of participants.

	NSI	VI^c^ Only	HI^c^ Only	DI^c^	P value^a^	Age adjusted P value^b^
N (Total = 215)	78	32	63	42		
**Mean age in years (SD) [range]**	68.17 (4.94) [59.7–80.3]	69.13 (5.30) [60.1–78.9]	72.20 (5.61) [60.0–80.3]	73.59 (4.60) [64.2–80.8]	<0.001	
Female, %	61.5	53.1	52.4	47.6	0.48	0.37
White, %	79.5	56.3	81.0	54.8	**<0.01**	0.17
Attained some college education, %	82.1	93.8	79.4	78.6	0.30	0.63
Number of comorbidities, %						
0	30.7	18.8	17.5	21.4	0.21	(ref)
1	35.9	18.8	36.5	23.8		0.8
2	16.7	31.3	25.4	28.6		0.3
3+	16.7	31.3	20.6	26.2		0.5
Currently married, %	76.9	65.6	77.8	66.7	0.38	0.30
Currently employed, %	43.6	46.9	28.6	38.1	0.22	0.85

^a^P value for age calculated by Analysis of Variance (ANOVA); Gender, Race, Education, Comorbidities, Marital Status, and Employment p values by Chi-squared tests.

^b^Age adjusted p values calculated by multinomial logistic regression.

^c^Vision Impairment was defined as visual field loss worse than -5 dB in the better-eye, Hearing Impairment was defined as pure tone average threshold worse than 25 dB in the better-ear, Dual Impairment was the presence of both vision and hearing impairments.

Abbreviations: NSI (No Sensory Impairment), VI (Vision Impairment), HI (Hearing Impairment), DI (Dual Impairment), SD (Standard Deviation), dB (decibels)

Note: Statistically significant (p<0.05) coefficients are shown in bold.

**Fig 1:**
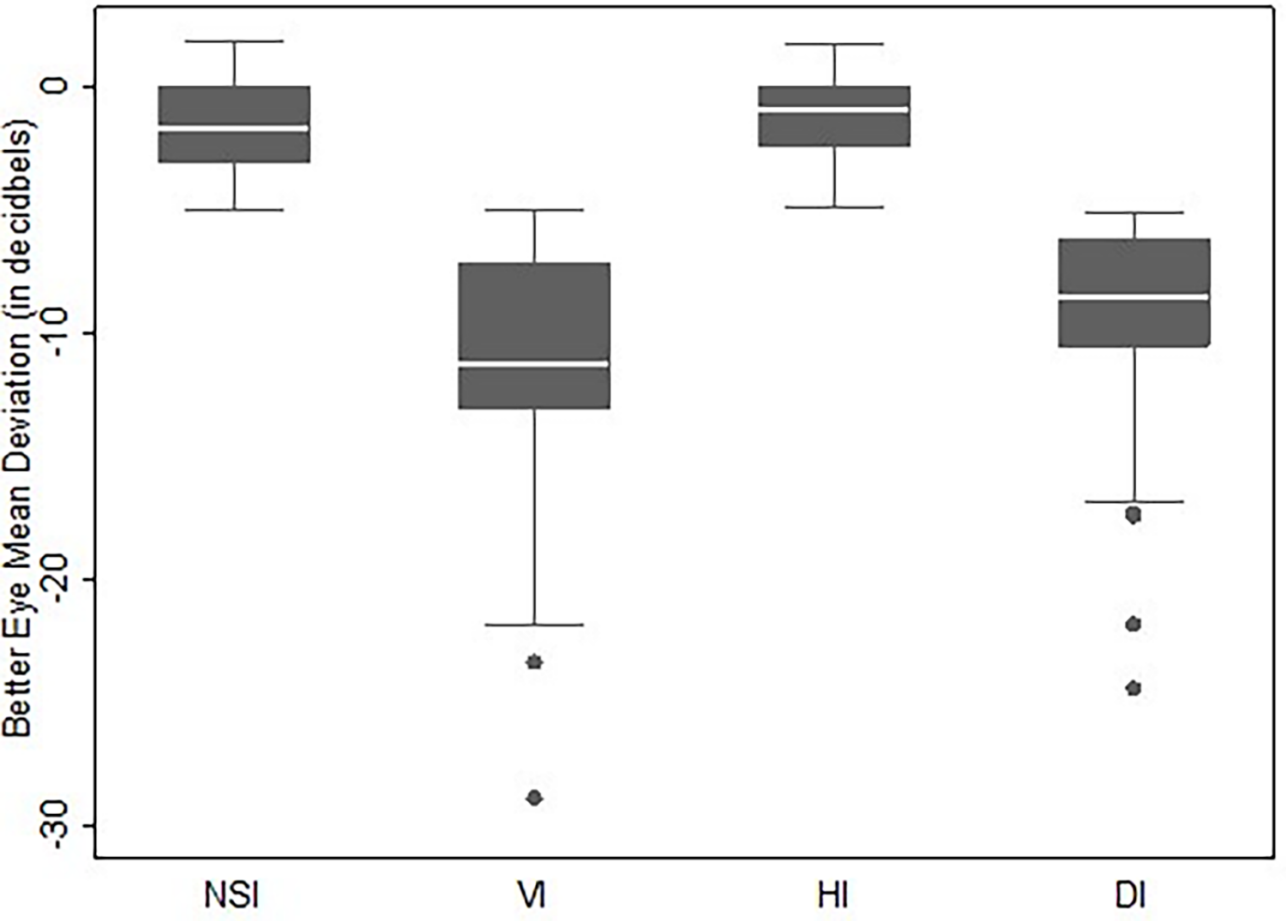
Better eye mean deviation (MD) in decibels on visual field testing. Abbreviations: NSI (No Sensory Impairment), VI (Vision Impairment), HI (Hearing Impairment), DI (Dual Impairment).

**Fig 2:**
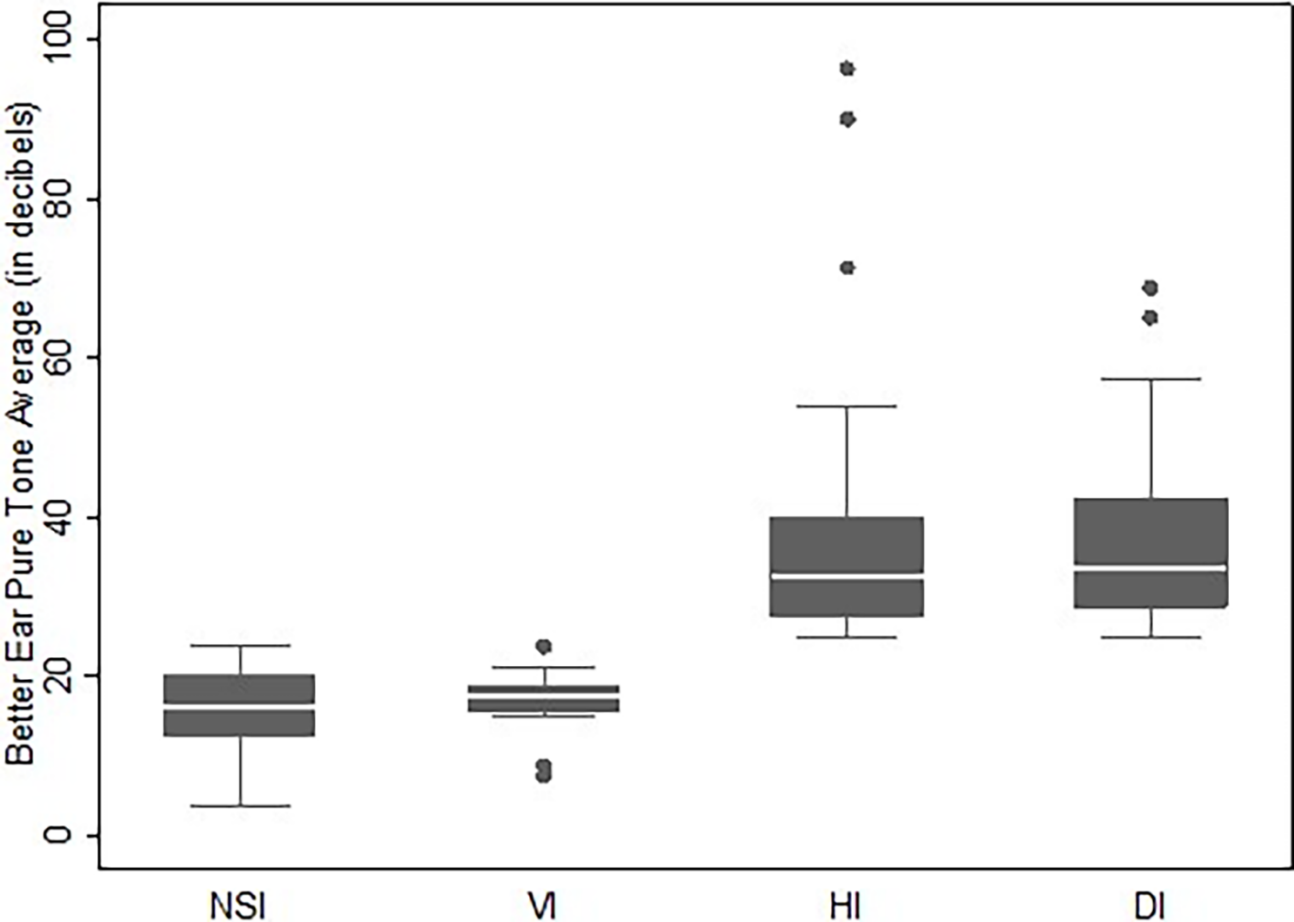
Better ear puretone average (PTA) threshold in decibels on audiometry. Abbreviations: NSI (No Sensory Impairment), VI (Vision Impairment), HI (Hearing Impairment), DI (Dual Impairment).

The average VF MD was not different between those with VI only and those with DI, -11.4 dB (SD: 5.5) and -9.7 dB (SD: 4.5) (p = 0.1), respectively. The mean PTA threshold was also similar between those with HI only and those with DI, 35.9 dB (SD: 13.3) and 36.9 dB (SD 10.8) (p = 0.67), respectively. The mean VA of the better eye was 20/25 for all four groups. **Figs 1 & 2**compare the range of better eye MD and better ear PTA threshold in dB across all four groups.

More DI participants (16.7%) reported moderate to extreme difficulty with driving, compared to 4.4% with no sensory impairment, 7.4% with VI only and 3.5% with HI only (**Table 2**, age-adjusted p = 0.04). More than 50% of the participants in each of DI and VI only groups reported any difficulty driving (age-adjusted p = 0.04), and 85.7% of the VI respondents and 83.3% of the DI participants reported vision related difficulty with driving (age-adjusted p = 0.4). More DI participants (7.7%) reported stopping driving due to difficulty with vision compared to no sensory impairment (1.4%), VI (3.3%) or HI alone (3.3%), however the difference was not statistically significant (age-adjusted p = 0.7).

**Table 2:** Participant self-reported functioning compared by sensory impairment.

	NSI (n = 78)	VI^c^ Only (n = 32)	HI^c^ Only (n = 63)	DI^c^ (n = 42)	P value^a^	Age adjusted P value^b^
**Physical Functioning**						
Mean (SD) grip strength in Kg	30.87 (9.62)	31.02 (9.59)	28.66 (8.75)	29.27 (9.42)	0.47	0.57
Severe difficulty with one or more ADLs, %	0.00	3.33	1.64	5.00	0.30	0.18
Severe difficulty with one or more IADLs, %	10.96	16.67	18.33	17.50	0.64	0.30
**Cognition and Mood**						
Mean (SD) MMblind score	21.23 (1.50)	21.60 (0.90)	21.10 (1.35)	20.70 (1.90)	0.08	0.33
Mean (SD) GDS score	1.17 (1.90)	1.67 (2.04)	1.24 (1.80)	1.63 (2.48)	0.53	0.62
**Self-reported Driving Limitation**						
Moderate to extreme difficulty driving, %	4.41	7.41	3.51	16.67	**<0.001**	**0.04**
Any difficulty driving, %	19.12	51.85	21.05	50.00	**<0.001**	**0.04**
**Driving limitation due to vision**, %	69.2	85.7	66.7	83.3	0.50	0.40
Stopped driving due to vision, %	1.4	3.3	3.3	7.7	0.40	0.70
**Communication and Social Functioning**						
Mean (SD) HHIES score	2.82 (5.63)	3.33 (4.34)	9.66 (9.20)	10.04 (10.47)	**<0.001**	**<0.001**
Severe limitations due to hearing loss on HHIES, %	1.37	0.00	8.47	14.63	**<0.001**	**<0.01**
Mean (SD) QDS score	6.64 (2.68)	6.67 (2.54)	9.16 (4.52)	9.07 (4.77)	**<0.001**	**<0.001**
Reporting difficulty communicating due to hearing, %	8.22	6.67	22.95	26.83	**0.01**	**0.01**
Mean (SD) UCLA Loneliness score	31.86 (8.66)	31.03 (7.70)	31.82 (8.22)	32.25 (10.83)	0.96	0.65
Mean (SD) SNI (social network diversity) score	5.86 (1.61)	5.70 (1.78)	6.12 (1.94)	5.85 (1.76)	0.72	0.59
Would like more social activity (on SOA-II), %	33.87	17.39	23.53	39.39	0.20	0.94

^a^Mean Grip Strength, MMblind, GDS, HHIES, QDS, UCLA, SNI score p values by ANOVA; all other p values by Chi-squared tests.

^b^Age adjusted p values by generalized linear models and multinomial logistic regression, as appropriate.

^c^Vision Impairment was defined as visual field loss worse than -5 dB in the better-eye, Hearing Impairment was defined as pure tone average threshold worse than 25 dB in the better-ear, Dual Impairment was the presence of both vision and hearing impairments.

Abbreviations: NSI (No Sensory Impairment), VI (Vision Impairment), HI (Hearing Impairment), DI (Dual Impairment), SD (Standard Deviation), Kg (Kilograms), ADL (Activities of Daily Living), IADL (Intermediate Activities of Daily Living), MMblind (Mini Mental State Examination for the Visually Impaired), GDS (Geriatric Depression Scale), HHIES (Hearing Handicap in the Elderly Screening Version), QDS (Quantified Denver Scale of Communication), UCLA (University of California Los Angles Loneliness Scale), SNI (Social Network Index), SOA-II (Second Supplement on Ageing Social Activity Component)

Note: Statistically significant (p<0.05) coefficients are shown in bold.

DI participants were also more likely to report severe limitation due to hearing loss on HHIES than those with HI alone, 14.6% vs. 8.5% respectively (**Table 2**, age-adjusted p <0.01). Likewise, participants with DI (26.8%) reported more communication difficulties on the QDS than those with no sensory impairment (8.2%), VI (6.7%) and HI (23.0%) (age-adjusted p = 0.01). Among DI participants, 39.4% responded that they would like more social activity, but this was not significantly different from the VI alone (17.4%) and HI alone (23.5%) participants (age-adjusted p = 0.9).

Physical functioning as assessed using grip test was similar across groups.

On regression modeling, after adjustment for age, gender, race and number of comorbidities, for participants with VI and DI, the proportional odds of reporting none to moderate, and moderate to severe driving limitation was greater than those with no sensory impairment (4.19, 95% confidence interval, CI: 1.58–11.14 and 4.72, 95% CI: 1.76–12.67, respectively) (**Table 3**). Participants with DI also showed the greatest proportional odds of reporting none to moderate and moderate to severe driving limitation due to vision difficulty (3.73, 95% CI: 1.19–11.70).

The average MMblind score was slightly better for participants with VI only (β = 0.54, 95% CI: -0.07–1.15) compared to those with no sensory impairment, while for participants with DI and HI only, the difference in MMblind score was slightly worse (β = -0.16, 95% CI: -0.73–0.43 and β = -0.03, 95% CI: -0.53–0.47, respectively) (**Table 3**), but these differences were not statistically significant.

**Table 3:** Multivariable regression models^a^.

	NSI (n = 78)	VI only^b^ (n = 32) β (95% CI)	P value	HI only^b^ (n = 63) β (95% CI)	P value	DI^b^ (n = 42) β (95% CI)	P value
**Physical Functioning**							
Difference in grip strength (in Kg)	(ref)	-0.52 (-3.04, 2.00)	0.68	**-2.27 (-4.38, -0.16)**	**0.03**	-2.02 (-4.47, 0.42)	0.10
Odds^c^ of any difficulty with one or more IADL	(ref)	1.40 (0.49, 3.97)	0.53	1.60 (0.67, 3.80)	0.29	1.76 (0.66, 4.71)	0.26
**Cognition and Mood**							
Difference in MMblind score	(ref)	0.54 (-0.07, 1.15)	0.08	-0.03 (-0.53, 0.47)	0.91	-0.16 (-0.73, 0.43)	0.06
Odds^c^ of depression	(ref)	2.05 (0.36, 11.78)	0.42	1.52 (0.30, 7.81)	0.62	1.17 (0.16, 8.36)	0.88
**Self-reported Driving Limitation**							
Proportional odds^c^ of moderate to severe driving difficulty	(ref)	**4.19 (1.58, 11.14)**	**<0.01**	1.01 (0.40, 2.56)	0.98	**4.72 (1.76, 12.67)**	**<0.01**
Proportional odds^c^ of moderate to severe driving limitation due to vision	(ref)	2.63 (0.78, 8.90)	0.12	1.03 (0.32, 3.30)	0.96	**3.73 (1.19, 11.70)**	**0.02**
**Communication and Social Functioning**							
Odds^c^ of reporting moderate to severe hearing handicap on HHIES	(ref)	**4.29 (1.07, 17.32)**	**0.04**	**13.90 (4.29, 45.09)**	**<0.001**	**15.42 (4.31, 55.22)**	**<0.001**
Odds^c^ of reporting difficulty on QDS	(ref)	0.80 (0.14, 4.47)	0.80	**3.41 (1.12, 10.38)**	**0.03**	**4.91 (1.44, 16.71)**	**0.01**
Odds^c^ of wanting more social activity (on SOA-II)	(ref)	0.30 (0.08, 1.12)	0.07	0.56 (0.23, 1.40)	0.22	1.10 (0.41, 2.97)	0.84

^a^Adjusted for age, gender, race, and number of comorbidities (none, 1, 2 or 3+).

^b^Vision Impairment was defined as visual field loss worse than -5 dB in the better-eye, Hearing Impairment was defined as pure tone average threshold worse than 25 dB in the better-ear, Dual Impairment was the presence of both vision and hearing impairments.

^c^Coefficients for logistic models have been exponentiated.

Abbreviations: NSI (No Sensory Impairment), VI (Vision Impairment), HI (Hearing Impairment), DI (Dual Impairment), SD (Standard Deviation), Kg (Kilograms), ADL (Activities of Daily Living), IADL (Intermediate Activities of Daily Living), MMblind (Mini Mental State Examination for the Visually Impaired), GDS (Geriatric Depression Scale), HHIES (Hearing Handicap in the Elderly Screening Version), QDS (Quantified Denver Scale of Communication), SOA-II (Second Supplement on Ageing Social Activity Component)

Note: Statistically significant (p<0.05) coefficients are shown in bold.

Compared to those with no sensory impairment, participants with VI had 4.29 (95% CI: 1.07–17.32) greater odds of reporting moderate to severe hearing handicap, while participants with HI had 13.90 (95% CI: 4.29–45.09) greater odds, and those with DI had 15.42 (95% CI: 4.31–55.22) greater odds (**Table 3**). Participants with HI and DI were also significantly more likely to report difficulty due to hearing on the QDS: for those with HI only, the odds of difficulty due to hearing on the QDS was 3.41 (1.12, 10.38) times greater than those with no sensory impairment, and for those with DI, the odds were 4.91 (1.44, 16.71) times greater than those with no sensory impairment.

Those with HI alone had lower grip strength than those with no sensory impairment (β = -2.27, 95% CI: -4.38, -0.16), while no differences were noted in the VI alone and DI groups.

## Discussion

This exploratory study assesses the combination of HI and VI based on VF loss, whereas prior studies of DI have generally used either VA or self-reported VI to define VI. It is important to note that VI participants in our study had excellent VA (a measure of central vision), and thus may not have met narrower definitions of VI. Although the mean VA of the better eye was 20/25 for all four groups, those with DI from glaucoma and HI reported generally poorer performance on most assessments of function. In particular, the magnitude of driving limitation and communication difficulties, was significantly greater for those with DI than for those with a single sensory impairment, and these findings are consistent with prior studies of DI.[17]

One of the strengths of our study is the ability to directly compare participants with VI from glaucoma to those with HI. Here, participants with HI were more likely to be older and White, and this is consistent with previous literature on presbycusis.[34] Cognition appeared to be similar between both groups, as were difficulties with ADLs. Not surprisingly, subjects with VI from glaucoma reported more difficulty driving than those with HI, and those with HI reported more verbal communication difficulties than those with VI from glaucoma, since each of these tasks depends primarily on a single sense (driving depends primarily on vision and verbal communication primarily on hearing). Subjects with VI from glaucoma were also less likely to report feeling socially isolated than HI participants. Although we were unable to find any prior studies comparing social isolation in glaucoma patients to those with HI, a prior study interviewed participants from 1994 National Health Interview Survey (NHIS) using the SOA-II and showed similar proportions of those with single and DI reporting they “would like to do more” social activity (33.7% for participants with HI and VI compared to 31.0% for those with VI only and 25.1% for those with no sensory impairment).[35]

We used self-reported questionnaires to determine functional status, thus all conclusions that we draw are inherently limited by the validity of the questionnaires. Further, since the questionnaires rely on self-report, the subjective nature of questions may have resulted in a measurement bias, for example, phrases such as “some of the time” or “moderate difficulty” may be interpreted differently by each participant which could lead to over- or underestimation of the association between sensory impairment and self-reported functional status. MMblind scores have been shown to vary by age, race and level of education.[36] In our study we adjusted scores for age and race, but not level of education since more than 70% of participants in each group had attained at least some college education. Although most participants had similar levels of education, it is possible that by not adjusting for education, MMblind scores may have been overestimated for some participants. Another limitation of the MMblind is that it accounts for VI but not HI, which is likely why in our study those with VI had slightly better MMblind scores than those with HI. Additionally, since the questionnaires were generally read aloud to participants, with accommodations in speaking volume and speed made for participants with hearing loss, this could have led to some bias in our measurements, especially for participants with hearing loss.

Despite the fact that we enrolled over 200 subjects, this was an exploratory study and our analyses were limited by relatively small numbers in each category of impairment. Although other measures of visual ability such as visual acuity and contrast sensitivity were measured, we were specifically interested in the effect of visual field loss, and given the already small numbers in glaucoma and non-glaucoma groups, we did not further divide them by VA or contrast sensitivity. Furthermore, the mean VA in the better eye was the same across all four groups. Standard definitions of *mild* VF VI (MD worse than -5 dB in the better eye)[37], and *mild* HI (PTA threshold greater than 25 dB in the better ear)[38] were used to dichotomize VI and HI. Although generally accepted definitions of VI and HI, it is possible that in using mild cut-points, the effect of sensory impairment on functional status may have been attenuated. However, setting the cutoffs to include only severe impairment did not result in changes in statistical inferences (data not shown). This is mostly likely because using severe definitions reduced number of participants in each sensory impairment group, therefore further reducing power and limiting our ability to see a significant differences in these analyses. Additionally, our study population comprised patients of whose primary spoken language was English, most of whom had completed some college education. Consequently, our results may not be generalizable to non-English speakers and those with lower education.

Overall, the results from this exploratory pilot study suggest that those with glaucoma and HI face challenges functioning day to day. Although they may have excellent visual acuity, older individuals with DI from glaucoma and HI appear to have significantly more limitation in driving and more difficulty with verbal communication than those with a single sensory impairment. Given the high prevalence of both glaucoma and HI in older adults, a larger study assessing the combined effect of VF loss and HI would be an important next step. Health professionals should include VF loss as a type of VI when assessing and managing individuals with DI.

## Supporting information

S1 File Study dataset. (DTA)
